# The interaction of hepatitis A virus (HAV) with soluble forms of its cellular receptor 1 (HAVCR1) share the physiological requirements of infectivity in cell culture

**DOI:** 10.1186/1743-422X-6-175

**Published:** 2009-10-27

**Authors:** Erica Silberstein, Krishnamurthy Konduru, Gerardo G Kaplan

**Affiliations:** 1 Laboratory of Hepatitis and Related Emerging Agents, Center for Biologics Evaluation and Research, Food and Drug Administration, 8800 Rockville Pike, Bethesda, MD 20892, USA

## Abstract

**Background:**

Hepatitis A virus (HAV), an atypical *Picornaviridae *that causes acute hepatitis in humans, usurps the HAV cellular receptor 1 (HAVCR1) to infect cells. HAVCR1 is a class 1 integral membrane glycoprotein that contains two extracellular domains: a virus-binding immunoglobulin-like (IgV) domain and a mucin-like domain that extends the IgV from the cell membrane. Soluble forms of HAVCR1 bind, alter, and neutralize cell culture-adapted HAV, which is attenuated for humans. However, the requirements of the HAV-HAVCR1 interaction have not been fully characterized, and it has not been determined whether HAVCR1 also serves as a receptor for wild-type (wt) HAV. Here, we used HAV soluble receptor neutralization and alteration assays to study the requirements of the HAV-HAVCR1 interaction and to determine whether HAVCR1 is also a receptor for wt HAV.

**Results:**

Treatment of HAV with a soluble form of HAVCR1 that contained the IgV and two-thirds of the mucin domain fused to the Fc fragment of human IgG1 (D1 muc-Fc), altered particles at 37°C but left a residual level of unaltered particles at 4°C. The kinetics of neutralization of HAV by D1 muc-Fc was faster at 37°C than at 4°C. Alteration of HAV particles by D1 muc-Fc required Ca, which could not be replaced by Li, Na, Mg, Mn, or Zn. Neutralization of HAV by D1 muc-Fc occurred at pH 5 to 8 but was more efficient at pH 6 to 7. D1 muc-Fc neutralized wt HAV as determined by a cell culture system that allows the growth of wt HAV.

**Conclusion:**

The interaction of HAV with soluble forms of HAVCR1 shares the temperature, Ca, and pH requirements for infectivity in cell culture and therefore mimics the cell entry process of HAV. Since soluble forms of HAVCR1 also neutralized wt HAV, this receptor may play a significant role in pathogenesis of HAV.

## Background

Hepatitis A virus (HAV), a *Picornaviridae *that causes acute hepatitis in humans, has a positive sense RNA genome of approximately 7.5 kb encapsidated in a shell formed by 60 copies of at least three viral proteins, VP1, VP2, and VP3 [1]. A small unmyristoylated VP4 of 23 amino acids is needed for capsid assembly [2] but has not been detected in mature virions. Nonstructural protein 2A remains associated with the structural proteins and serves as a signal for the assembly of pentamers, which are precursors involved in the morphogenesis of the capsid [2].

The cell entry mechanism of HAV is poorly understood and cannot be inferred from other picornaviruses due to its atypical characteristics and the diverse entry modes used by other members of the family. HAV infects African green monkey kidney cells via the hepatitis A cellular receptor 1 (HAVCR1) [3]. The ectodomain of HAVCR1 contains an N-terminal immunoglobulin-like (Ig-like) region (IgV), followed by an O-glycosylated threonine, serine, and proline-rich mucin-like region that extends the IgV well above the cell surface. The monkey and human HAVCR1 share 79% homology and have HAV-receptor function [3,4]. HAVCR1 is a costimulatory receptor in T cells and has been implicated as an asthma determinant [5,6]. HAV infection has a protective effect in the development of allergy and asthma [7,8] but the role of HAVCR1 in this protective affect is not well understood and will require a detailed analysis of the virus-receptor interaction. We have previously shown that the IgV domain of HAVCR1 is necessary for HAV receptor function [9] and that the IgV and mucin domains are required to induce conformational changes leading to the uncoating of HAV [10]. Some of the requirements for the interaction of HAV with cells were defined by experiments of classical virology [11,12]. However, the development of a comprehensive cell entry model for HAV will require further investigation. In this paper, we study the influence of temperature, cations, and pH in the interaction of HAV with soluble HAVCR1 and found that it shares the physiological requirements for infectivity in cell culture. Here, we also showed that wild-type (wt) HAV interacts with HAVCR1, which suggested that this receptor may play a critical role in pathogenesis of HAV.

## Results

### Soluble HAVCR1 neutralized HAV at 4 and 37°C

**Figure 1:**
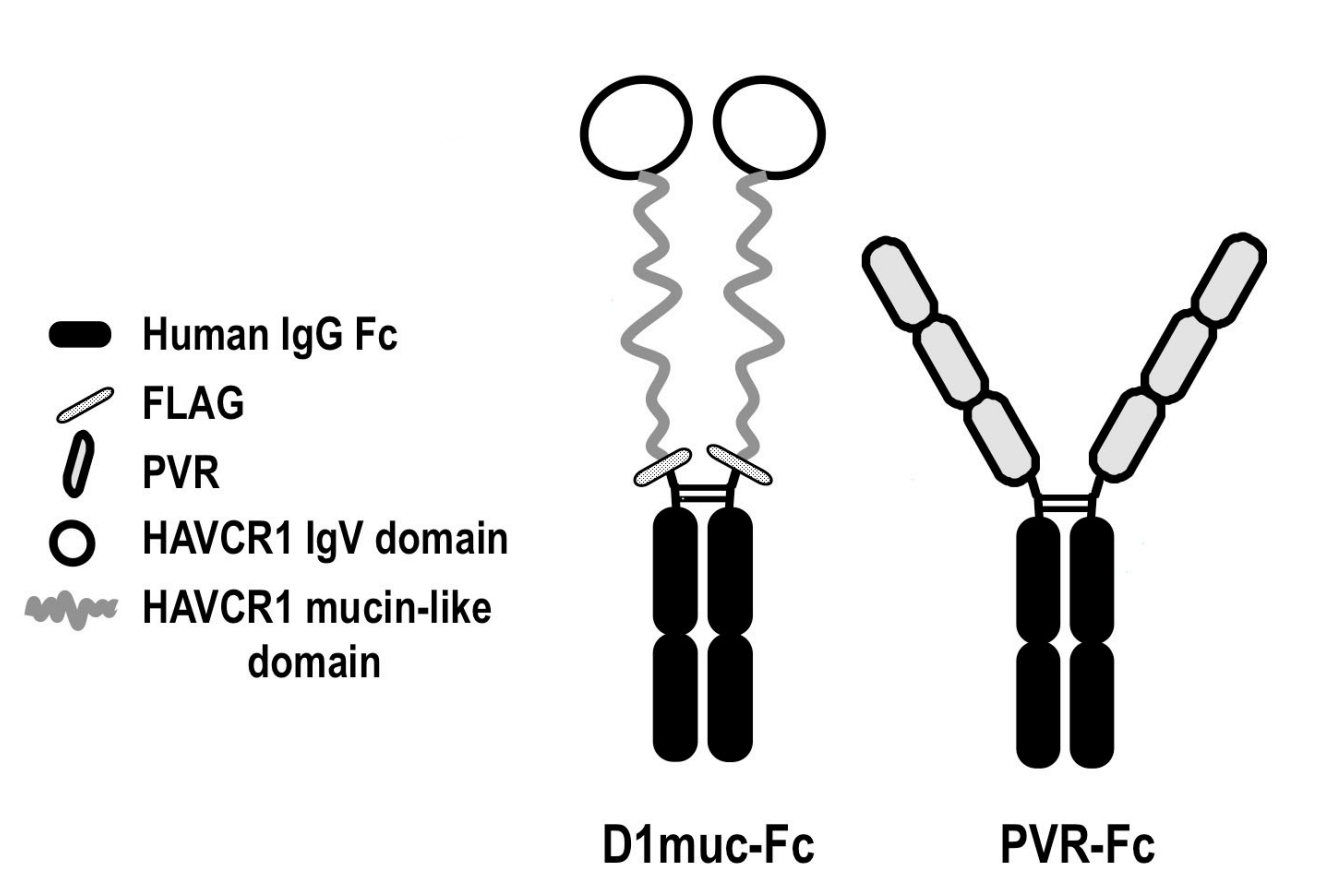
**Schematic representation of Fc fusion proteins**. D1muc-Fc is a soluble form of HAVCR1 containing the IgV and two-thirds of the mucin-like domain of HAVCR1 fused to the hinge and Fc regions of human IgG1. A FLAG tag consisting of 8 amino acid residues was introduced between the HAVCR1 and hinge portions. PVR-Fc, a soluble receptor form containing the whole ectodomain of the poliovirus receptor fused to the same hinge and Fc fragment of human IgG1 was used as a negative control fusion protein. Construction and purification of D1muc-Fc and PVR-Fc was done as described [17].

**Figure 2:**
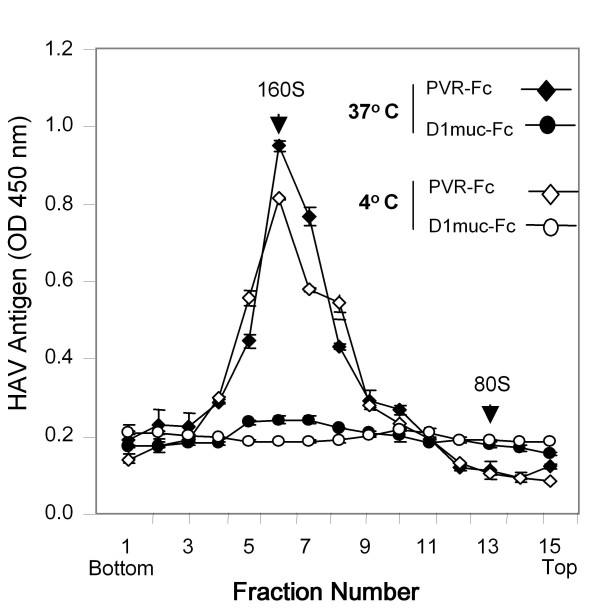
**Effect of temperature in the alteration of HAV by soluble HAVCR1**. Sedimentation analysis of HAV particles treated with soluble receptors at different temperatures. Purified HAV virions were incubated with 20 μg of D1 muc-Fc or control PVR-Fc for 30 min at 4°C or 37°C, loaded onto linear 15 to 30% sucrose gradients, and ultracentrifuged. The gradient was collected from the bottom in 20 fractions, and HAV antigen in the fractions was determined by ELISA [17]. The first 15 fractions of each gradient are shown. Data are mean results from duplicate wells; duplicate values varied by less than 10%. Poliovirus native virions and empty particles labeled with ^35^S-methionine were used as 160S and 80S sedimentation markers. The top and bottom of the gradients are indicated.

**Figure 3:**
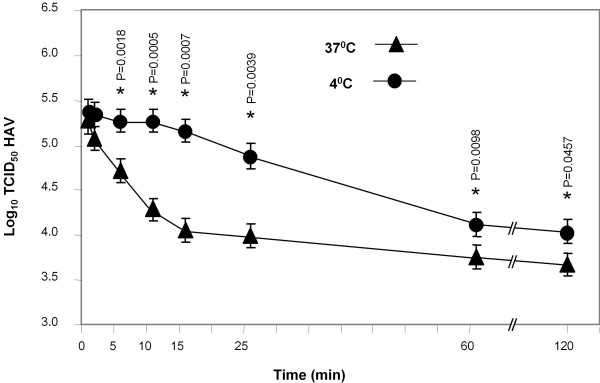
**Effect of temperature in the neutralization of HAV by soluble HAVCR1**. Kinetics of neutralization of cell culture-adapted HAV by soluble HAVCR1 at different temperatures. HAV(10^6 ^TCID_50_) was incubated with 20 μg of purified D1 muc-Fc or control PVR-Fc, for different times at 4°C and 37°C. After incubation, neutralization reactions were diluted 1/100 and residual infectious HAV was titrated by an ELISA end point dilution assay in 96-well plates containing confluent monolayers of GL37 cells. Values are the log_10 _TCID_50_/ml of HAV determined by the Reed and Muench method [30] and the standard deviations are shown as error bars. Significant differences (P < 0.05) in viral titers at 37°C and 4°C for each time point are indicated with asterisks and their corresponding p-values.

We previously showed that D1 muc-Fc (Figure 1), a Fc fusion protein containing the IgV and two-thirds of the mucin-like regions of HAVCR1, bound and altered HAV particles [13]. PVR-Fc, a similar Fc fusion protein containing the ectodomain of the poliovirus receptor PVR, did not interact with HAV [13]. To study the effect of temperature on the virus-receptor interaction, we treated HAV particles with D1 muc-Fc or PVR-Fc at 4°C or 37°C and analyzed their sedimentation profile in 15-30% sucrose gradients. After ultracentrifugation at 40,000 rpm in an SW40 rotor for 100 min at 4°C, we collected fractions from the bottom of the gradients and determined the sedimentation profile of the HAV particles by ELISA using anti-HAV antibodies [10]. Poliovirus virions (160S) and empty particles (80S) labeled *in vivo *with ^35^S-methionine were run in parallel gradients as sedimentation markers. Treatment of HAV with D1 muc-Fc at both temperatures altered the particles (Figure 2) whereas treatment with PVR-Fc did not affect the sedimentation of the virions. A residual level of virions treated with D1 muc-Fc at 4°C remained unaltered, which suggested that HAV-HAVCR1 interaction at this low temperature was less efficient than at 37°C. To further evaluate the effect of temperature in the virus-receptor interaction, we analyzed the kinetics of neutralization of HAV by D1 muc-Fc at 4°C or 37°C using an ELISA endpoint dilution assay in 96-well plates containing AGMK GL37 cells. Briefly, 10^6 ^TCID_50 _of HAV were incubated at 4°C or 37°C with 20 μg of D1 muc-Fc or PVR-Fc and the reduction in the HAV titers was evaluated at different incubation times. Figure 3 shows that the kinetics of neutralization was faster at 37°C than at 4°C, and that viral titers were significantly lower after 5 min of incubation at 37°C than at 4°C (p < 0.05). At 37°C, D1 muc-Fc neutralized approximately 1.3 log of HAV during the first 15 min of incubation. The rate of neutralization diminished dramatically after 15 min, with an additional 0.5 log of HAV neutralized after 2 h incubation. At 4°C, the HAV titers remained stable during the first 15 min incubation and then decreased slowly about 1 log in the following 45 min. HAV neutralization did not increase significantly between 1 h and 2 h incubation (p > 0.05), but viral titers were about 0.5 log lower at 37°C than at 4°C. As expected, treatment with negative control PVR-Fc at 4°C or 37°C did not affect the HAV titers (data not shown). These data showed a dramatic difference in the kinetics of interaction of HAV with HAVCR1 at 4°C and 37°C and indicated that the virus-receptor interaction is faster and more efficient at physiological temperature.

### Alteration of HAV by D1 muc-Fc is a calcium-dependent process

**Figure 4:**
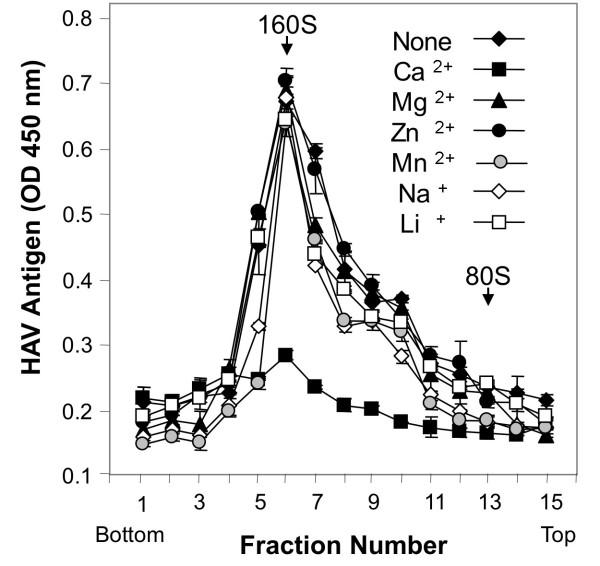
**Effect of monovalent and divalent cations in the alteration of HAV by soluble HAVCR1**. Sedimentation analysis of cell culture-adapted HAV treated with soluble HAVCR1 in the presence or absence of different cations. Purified HAV virions were incubated with 5 μg of D1 muc-Fc for 2 h at 37°C in the presence of 1 mM CaCl_2_, 10 mM MgCl_2_, 0.1 mM ZnCl_2_, 0.1 μM MnCl_2_, 500 mM NaCl, or 10 mM LiCl and also in the absence of any cations, and sedimented in linear 15 to 30% sucrose gradients as described in Figure 2. HAV antigen was detected by ELISA. The data are mean OD 450 nm from duplicate wells; duplicate values varied by less than 10%. Poliovirus native virions and empty particles labeled with ^35^S-methionine were used as 160S and 80S sedimentation markers.

**Figure 5:**
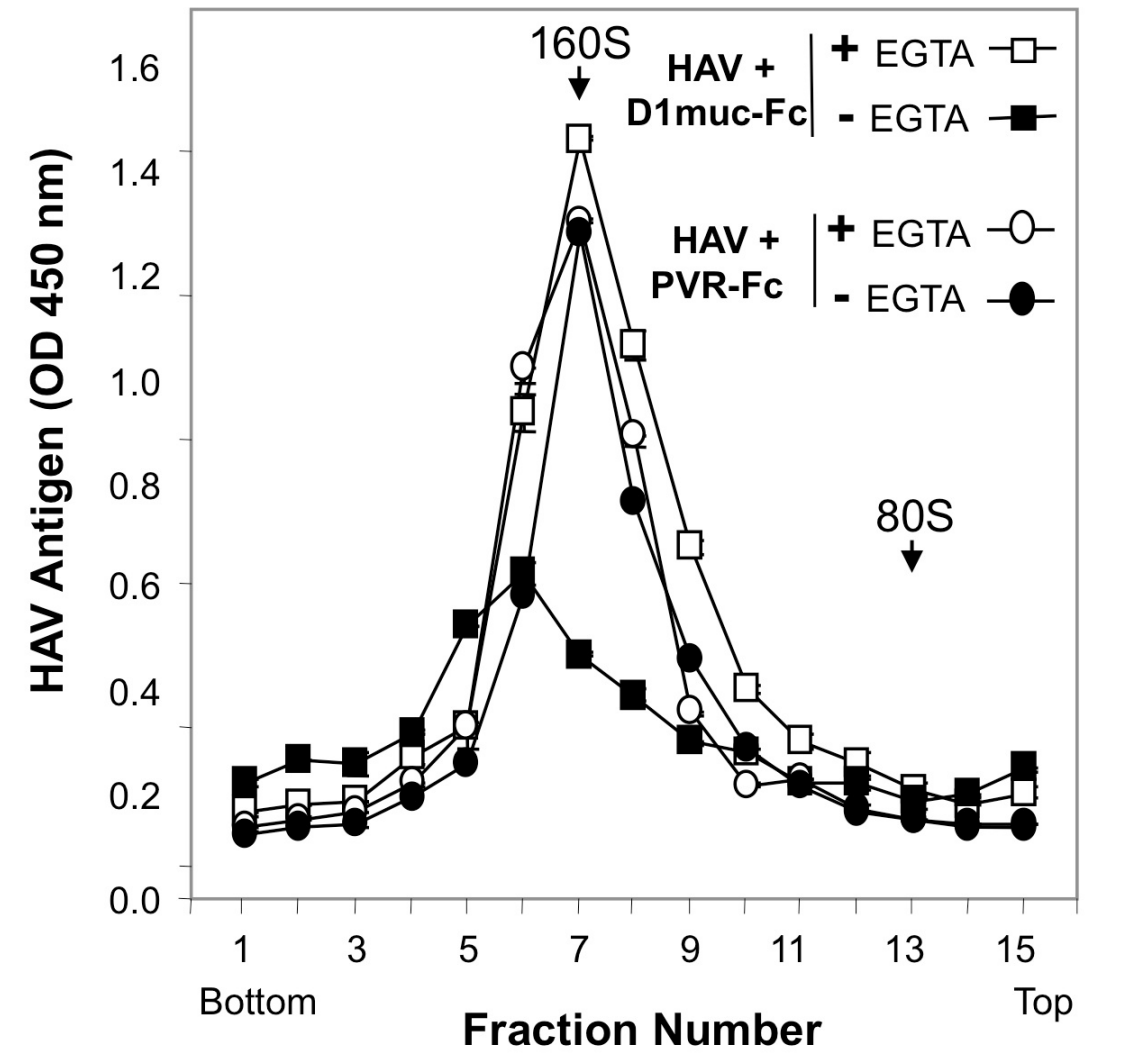
**EGTA inhibits the alteration of HAV by soluble HAVCR1**. Sedimentation analysis of HAV alteration by soluble HAVCR1 in the presence of ethylene glycol bis(beta-aminoethyl ether)-N, N, N', N'-tetraacetic acid (EGTA). Purified HAV virions were incubated with 5 μg of D1 muc-Fc or PVR-Fc for 2 h at 37°C with or without the addition of EGTA and separated by sedimentation in linear 15 to 30% sucrose gradients as described in Figure 2. The HAV antigen in the gradient fractions was detected by ELISA. Data are the mean OD 450 nm from duplicate wells; duplicate values varied by less than 10%. Poliovirus native virions and empty particles labeled with ^35^S-methionine were used as 160S and 80S sedimentation markers.

Since attachment and infectivity of HAV in cell culture is a Ca-dependent process [12,14,15], we analyzed the cation requirement of the interaction of HAV with soluble forms of HAVCR1. Purified HAV virions were treated with D1 muc-Fc for 2 h at 37°C in the presence of 1 mM CaCl_2_, 10 mM MgCl_2_, 0.1 mM ZnCl_2_, 0.1 μM MnCl_2_, 500 mM NaCl, or 10 mM LiCl and in the absence of any cations. The effect of the different treatments was studied by sedimentation in sucrose gradients. HAV particles were altered only in the presence of CaCl_2 _(Figure 4), which mimics the divalent cation requirement for infectivity of HAV in cell culture. In the absence of D1 muc-Fc, treatment with 1 mM CaCl_2 _did not alter HAV particles showing that the virions were stable at this concentration of Ca ions (data not shown). To further confirm the Ca dependency, we tested the effect of the Ca chelating agent ethylene glycol bis(beta-aminoethyl ether)-N, N, N', N'-tetraacetic acid (EGTA) in the alteration of the HAV particles. Purified HAV particles were treated with 5 μg of D1 muc-Fc or control PVR-Fc in a buffer containing 1 mM CaCl_2 _in the presence or absence of 2 mM EGTA. After 2 h incubation at 37°C, the HAV particles were analyzed by sedimentation in 15 to 30% sucrose gradients (Figure 5). The sedimentation profiles revealed that the EGTA treatment inhibited the alteration of the HAV particles. Treatment of HAV with PVR-Fc in the presence of EGTA did not affect the sedimentation of the virions, which indicated that EGTA did not affect the stability of the HAV particles. Our results clearly showed that the alteration of HAV particles by D1 muc-Fc required the presence of Ca ions.

### The HAV-HAVCR1 interaction is optimal at neutral pH

**Figure 6:**
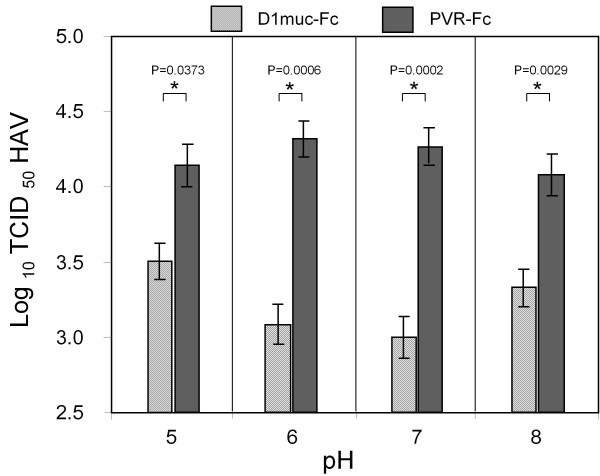
**Effect of pH on soluble HAVCR1-mediated neutralization of HAV**. HAV PI (10^5 ^TCID_50_) was incubated with 20 μg of purified D1 muc-Fc (hatched bars) or control PVR-Fc (closed bars) in EMEM at pH 5, 6, 7, or 8 overnight at 4°C. Residual infectious HAV was titrated by an ELISA end point dilution assay. Values are the log_10 _TCID_50 _HAV determined by the Reed and Muench method [30] and the standard deviations are shown as error bars. Significant differences (p < 0.05) in titers of virus treated with D1 muc-Fc or PVR-Fc at each pH are indicated with asterisks and their corresponding p-values.

To analyze the pH requirements for the virus-receptor interaction, HAV (10^5 ^TCID_50_) was treated overnight with 20 μg of purified D1 muc-Fc or control PVR-Fc at pH 5, 6, 7 or 8, and residual HAV infectivity was titrated by an ELISA endpoint dilution assay (Figure 6). D1 muc-Fc neutralized HAV at all pHs but maximum levels of neutralization were achieved at pH 6-7. Treatment of HAV with PVR-Fc at the different pHs did not significantly affect the viral titers, which showed that HAV was stable at these experimental conditions. Therefore, D1 muc-Fc neutralized HAV in a wide pH range (from 5 to 8) with an optimal interaction at pH 7.

### Neutralization of wt HAV by soluble HAVCR1

**Figure 7:**
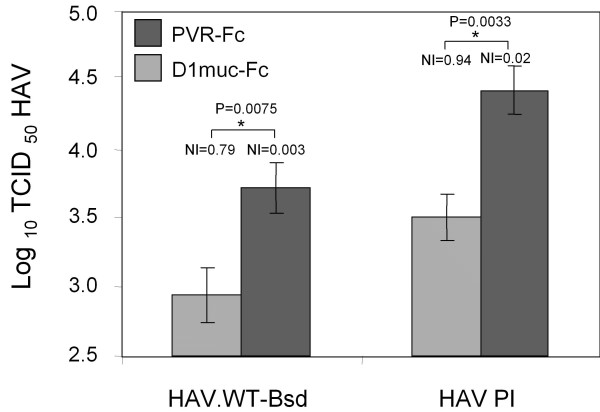
**Neutralization of wild-type HAV by soluble HAVCR1**. HAV.WT-Bsd (5 × 10^5 ^TCID_50_), a wild-type HAV recombinant virus containing a blasticidin selectable marker, or cell culture-adapted HAV (5 × 10^6^TCID_50_) were incubated with 20 μg of D1 muc-Fc (hatched bars) or PVR-Fc (closed bars) for 2 h at 37°C. Residual infectious wt HAV virus was titrated by the antibiotic resistance titration assay (ARTA) in Huh-7 AI in the presence of 2 μg/ml blasticidin. Residual infectious cell culture-adapted HAV was titrated by an ELISA end point dilution assay [16]. Values are the log_10 _TCID_50 _of HAV determined by the Reed and Muench method [30], and the standard deviations are shown as error bars. The neutralization index (NI) of soluble receptor-treated virus compared to mock-treated virus is indicated above each bar. Very significant differences (p < 0.01) in titers of virus treated with D1 muc-Fc or PVR-Fc are indicated with asterisks and their corresponding p-values.

Cell culture-adapted strains of HAV are attenuated for humans and contain several mutations, including at least two located in the viral capsid proteins VP1 and VP2. We have shown that soluble HAVCR1 neutralize the cell culture-adapted strain HM175 of HAV [16,17]. To analyze whether HAVCR1 could also interact with wt HAV, we neutralized a wt HAV construct containing a blasticidin selectable marker (HAV.WT-Bsd) [18] with soluble D1 muc-Fc. Briefly, HAV.WT-Bsd (10^5^TCID_50_) was treated with 20 μg of D1 muc-Fc or PVR-Fc for 2 h at 37°C, and residual infectious virus was titrated in 96-well plates containing 20-50% confluent monolayers of Huh-7-A-I cells in the presence of 2 μg/ml blasticidin. At 7 days postinfection, viral titers were determined by assessing the presence of blasticidin-resistant surviving cells in replica wells [19]. Figure 7 shows that D1 muc-Fc neutralized approximately 1 log of HAV.WT-Bsd (NI = 0.79) whereas control PVR-Fc had almost no effect in the viral titer (NI = 0.003). This very significant reduction in infectivity (p < 0.01) indicated that HAVCR1 also functions as a receptor for wt HAV. Treatment of cell culture adapted HAV with D1 muc-Fc under the same conditions also reduced infectivity approximately 1 log (NI = 0.94) whereas PVR-Fc had almost no effect (NI = 0.02), which indicated that HAVCR1 interacted efficiently with wt HAV.

## Discussion

We have previously determined that the IgV domain of HAVCR1 is required for HAV-receptor function [9,16]. We also showed that the IgV and the mucin-like regions are needed to induce conformational changes leading to the uncoating of HAV particles [17]. To further characterize how HAV interacts with HAVCR1, we evaluated the temperature, cation, and pH requirements of the HAVCR1-mediated alteration and neutralization of HAV. Soluble HAVCR1 neutralized HAV at 37°C and 4°C but with significantly different kinetics (Figure 3). Our data are consistent with previous studies showing a faster kinetics of HAV binding and infection at 37°C than at 4°C [20-22]. The difference in the neutralization kinetics at 37°C and 4°C indicated that the neutralization reaction followed different mechanisms. At 37°C, the neutralization reaction followed a one-hit kinetics, in which only one or few receptor molecules per particle were needed to neutralize the virus [23,24]. At 4°C, it most likely followed a multi-hit kinetics, in which D1 muc-Fc bound slowly to HAV covering the particles and triggering neutralization after saturation of the binding sites. Alternatively, binding of the soluble receptors at 4°C may induce the slow diffusion of a putative stabilizing factor that triggered the destabilization of the viral particles.

Our studies showed that the alteration of HAV by soluble HAVCR1 is a Ca-dependent process (Figure 4 and 5), which is consistent with previously published reports showing that binding of HAV to cells and infection is enhanced by Ca ions [20-22]. Calcium plays an important role at different levels of the cell entry process of viruses [25]. Since Ca is required for the HAV-HAVCR1 interaction, it is possible that Ca ions enhance binding of HAV to HAVCR1 and allow the alteration of the viral particles, a process required to deliver the virus genome into the cytoplasm. It has been previously shown that Ca can slowly destabilize the HAV particles [20-22], so Ca ions could be required to alter the virions after binding to HAVCR1. Alternatively, the mucin-like domain of HAVCR1 may require calcium ions for the proper presentation of the IgV domain to allow virus binding and/or alteration. Further research will be required to understand the exact role of Ca ions in the HAV-HAVCR1 interaction.

Soluble HAVCR1 neutralized HAV at pH 5 to 8 but the reaction proceeded more efficiently at neutral pH (Figure 6). HAV is transmitted through the fecal-oral route, so the ability of HAV to interact with HAVCR1 in a wide pH range suggested that HAVCR1 could mediate infection of cells in the gastrointestinal track. The ability of HAVCR1 to interact with HAV at different pHs may also have a role in the cell entry process of HAV. Although the uncoating site of HAV is not know, the efficient virus-receptor interaction at low pH also suggested that HAV could be internalized to a low pH endocytic compartment prior to delivery of the RNA genome to the cytoplasm. Since low pH enhanced attachment HAV to BS-C1 cells [20] and red blood cells [26] but did not increase neutralization by soluble HAVCR1, it is possible that at low pH the virus particles expose additional epitopes or enhanced binding sites that allow attachment to other receptors at the cell surface.

The role of HAVCR1 in pathogenesis of HAV has been difficult to determine due to the lack of a small animal model for HAV. HAVCR1 was identified as a cellular receptor for cell culture adapted strains of HAV [3,4] in cell culture. Cell culture-adapted strains of HAV contain several mutations that attenuate the virus and could potentially change the receptor preference of the virus. Our finding that soluble HAVCR1 neutralized wt HAV suggested that this virus could use HAVCR1 to cause disease in humans.

## Conclusion

Our results clearly show that the interaction of HAV with soluble HAVCR1 mimics the cell entry process of HAV. We previously showed that soluble forms of HAVCR1 altered the viral particles, a process required for uncoating of the viral genome. Here, we determined that neutralization and alteration of HAV by soluble HAVCR1 is a Ca-dependent process that is optimum at 37°C and occurs at pH 5 to 8, which are known requirements for HAV infectivity in cell culture. We also showed that HAVCR1 could function as cellular receptors for wt HAV since treatment with soluble receptors neutralized the virus. Taken together, our data suggested that HAVCR1 may play a significant role in pathogenesis of HAV.

## Methods

### Antisera

Anti-HAV serum was produced in rabbits immunized with commercially available HAV vaccine [16]. Phosphatase-labeled goat anti-rabbit IgG antibody was used as suggested by the manufacturer (Kirkegaard & Perry Laboratories, Inc.).

### Cells and viruses

The continuous clone GL37 of African green monkey kidney (AGMK GL37) cells [27] was grown in Eagle's minimal essential medium (EMEM) containing 10% heat-inactivated fetal bovine serum (FBS).

Chinese hamster ovary (CHO) cell transfectants expressing Fc fusion proteins of HAVCR1 or the poliovirus receptor (PVR) were grown in Iscove's medium containing 10% dialyzed FBS and 5 mM methotrexate (MP Biochemicals) as described [16].

Huh-7-A-I cells, a clone of human hepatoma Huh-7 cells that allowed the stable growth of wt HAV [18], was grown in Dulbecco's modified minimal essential medium (DMEM) supplemented with 10% heat-inactivated FBS.

The cell culture-adapted strain HM175 of HAV was derived from infectious cDNA [28] and passed approximately 100 times in the continuous BSC-1 cell line of African green monkey kidney. Virus stocks were prepared by growing HAV in fetal Rhesus kidney (FRhK-4) cells for 10 days. Infected monolayers and culture supernatants were subjected to three freeze-and-thaw cycles, clarified by centrifugation, and stored at -70°C.

Purified HAV was produced in FRhK4 cells infected with a cytopathic variant of cell culture-adapted strain HM175 of HAV [29].

A wt HAV construct containing a blasticidin selectable marker cloned at the 2A-2B junction of wt HAV genome (HAV.WT-Bsd) was grown in Huh-7-A-I cells [18].

### HAV titer determination

Cell culture-adapted HAV was titrated by an endpoint dilution assay in 96-well plates containing confluent monolayers of AGMK GL37 cells [16]. Briefly, 8 to 16 replicate wells were inoculated with 100 μl each of 10-fold dilutions of HAV and grown at 35°C for 2 weeks in a CO_2 _incubator. Cells were fixed with 95% methanol, and HAV was detected by ELISA staining with rabbit anti-HAV antibody and peroxidase-labeled goat anti-rabbit antibody. Wells that developed at least 2.5 times the color of uninfected control wells were considered positive, and viral titers were calculated by the Reed and Muench method [30].

HAV.WT-Bsd was titrated using an antibiotic-resistance titration assay (ARTA) [19] based on the endpoint dilution of the virus in 96-well plates containing 20-50% confluent monolayers of Huh-7-A-I cells and the selection of antibiotic-resistant infected cells in the presence of 2 μg/ml blasticidin. Five to seven days after infection, the 96-well plates were examined under the microscope to determine the presence of live cells. Viral titers were determined using the Reed and Muench method [30]. The neutralization index (NI) was calculated with the formula NI = log (TCID_50 _mock-treated virus sample) - log(TCID_50 _receptor-treated virus sample) [31].

### Neutralization assays

HAV was neutralized with a purified recombinant proteins containing the IgV domain and 2/3 of the mucin-like region of HAVCR1 fused to the hinge and Fc portions of human IgG1 (D1 muc-Fc) [17]. A recombinant protein containing the ectodomain of PVR fused to the same Fc fragment (PVR-Fc) was used as a negative neutralization control [17].

To evaluate the effect of temperature in the virus -receptor interaction, cell culture-adapted HAV (5 × 10^6 ^TCID_50_) was incubated at 4°C or 37°C with 20 μg of D1 muc-Fc or PVR-Fc. Aliquots of the neutralization reaction were taken at different times after incubation. Samples were diluted 1/100 in cell culture media and residual infectious virus was titrated in 96-well plates containing confluent monolayers of the AGMK GL37 cells. The virus was inoculated in 8 to16 wells/dilution and adsorbed for 4 h at 37°C in a CO_2 _incubator. After washing three times with EMEM (to remove unbound virus), 200 μl/well of EMEM-10% FBS was added and the plates were incubated at 35°C under CO_2 _for 14 days, and HAV titers were determined by ELISA.

To neutralize HAV.WT-Bsd, 20 μg of D1 muc-Fc or PVR-Fc were incubated with 5 × 10^5 ^TCID_50 _of virus for 2 h at 37°C. Neutralization reactions were diluted 1/100 and residual infectious virus was titrated by ARTA in 96-well plates containing 20-50% confluent monolayers of Huh-7-AI cells. Viral titers were determined seven days after infection [18].

### Sedimentation analysis of HAV particles

HAV virions were purified by sedimentation in linear 15 to 30% sucrose gradients with a Beckman SW40 rotor at 4°C for 100 min at 40,000 rpm. Gradients were collected from the bottom in 20 fractions of 0.5 ml each, and HAV was detected by ELISA [16]. The 160S virion peak was pooled and stored at -70°C.

To study the effect of temperature in the alteration of HAV, sucrose-purified virions were treated with 20 μg of PVR-Fc or D1 muc-Fc for two hours at 4°C or 37°C, and analyzed by ultracentrifugation in 15 to 30% sucrose gradients as indicated above. To determine the requirement of monovalent or divalent cations for the alteration of HAV by soluble receptors, sucrose-purified virions were treated with 5 μg of PVR-Fc or D1 muc-Fc for 2 h at 4°C in the presence of 1 mM CaCl_2_, 10 mM MgCl_2_, 0.1 mM ZnCl_2_, 0.1 mM MnCl_2_, 500 mM NaCl or 10 mM LiCl. The sedimentation profile of HAV was analyzed by ultracentrifugation in 15 to 30% sucrose gradients as indicated above. ^35^S-labeled poliovirus [32] was used as a sedimentation marker, and 160S virions and 80S empty particles were identified by scintillation counting.

### Statistical analysis

Viral titers and standard deviations were determined by the Reed and Muench [30] method and calculated with the ID50 program developed by John L. Spouge (National Center for Biotechnology Information, NIH). Statistical significance between two viral titers was determined by the unpaired Student's t-test an calculated using Graph Pad software, and p-values were included in the text and figures.

## Competing interests

The authors declare that they have no competing interests.

The findings and conclusions in this article have not been formally disseminated by the Food and Drug Administration and should not be construed to represent any Agency determination or policy.

## Authors' contributions

ES and KK carried out the virology studies. ES, KK and GGK participated in the design of the study. GGK conceived and coordinated the study. ES, KK and GGK drafted the manuscript. All Authors read and approved the final manuscript.
